# Neuromuscular training for preventing ankle joint injuries in athletes: a systematic review and meta-analysis

**DOI:** 10.3389/fphys.2026.1854319

**Published:** 2026-07-22

**Authors:** Hexuan Xiang, Kexiang Yang, Huiling Liao, Xiaolong Zhang, Yunong Zhang, Hyun-Chul Jeong

**Affiliations:** 1 Department of Basic Education, Bijie Vocational and Technical College, Bijie, China; 2 Department of Physical Education, Jeonbuk National University, Jeonju, Republic of Korea; 3 Department of Physical Education, Sejong University, Seoul, Republic of Korea

**Keywords:** ankle joint injuries, Cumberland Ankle Instability Tool, foot and ankle ability measure, meta-analysis, neuromuscular training

## Abstract

Ankle joint injuries are among the most common sports-related injuries. However, determining the most effective training method to prevent these injuries remains a challenge. Neuromuscular training (NMT) is a preventive strategy aimed at reducing muscle and ligament injuries, yet its effectiveness in preventing ankle joint injuries is not fully understood, and robust clinical evidence is lacking. This systematic review and meta-analysis aim to summarize the preventive effects of NMT on ankle joint injuries and provide an overview of effective exercise components. To investigate the impact of neuromuscular training on the incidence of ankle joint injuries, dynamic neuromuscular control, foot and ankle functionality, and postural stability in athletes. A comprehensive search was conducted in Web of Science, PubMed, Cochrane Library, Google Scholar, and Embase databases for randomized controlled trials (RCTs) related to the use of neuromuscular training for the prevention of primary and secondary ankle joint injuries in athletes. The search was limited to studies published up until July 31, 2024. Relevant references from the included studies were also reviewed to identify additional literature. Two independent reviewers performed the search, selection, and data extraction. The risk of bias was assessed independently using the Cochrane Handbook. Meta-analyses were performed using Review Manager5.3. The quality of evidence was evaluated using the GRADE methodology. A total of 13 randomized controlled trials, involving 1, 476 participants, were included in the final analysis. Compared to no training or conventional training, neuromuscular training was associated with a 55% reduction in the incidence of ankle joint injuries (RR: 0.45, 95%CI: 0. 25-0. 81, I²: 62%, P: 0.007). In terms of dynamic neuromuscular control, NMT significantly increased reach distances in the Star Excursion Balance Test, specifically in the posteromedial direction (MD: 5.02 cm, 95%CI: 2.22-7. 81, I²: 1%, P: 0.0004), posterolateral direction (MD: 3.05 cm, 95%CI: 0.74-5.35, I²: 0%, P: 0.01), and anterior direction (MD: 2.23 cm, 95%CI: 0.05-4.40, I²: 0%, P: 0.05). Additionally, NMT improved foot and ankle function in the sports domain, as evidenced by an increased score in the Foot and Ankle Ability Measure (FAAM-Sports) (MD: 8.66, 95%CI: 5.10-12.21, I²: 0%, P: <0.00001). Descriptive analysis also suggested improvements in postural stability and ankle instability. However, there was no significant improvement in the foot and ankle function in the daily living domain (FAAM-ADL) (MD: 2.86, 95%CI: -2.20-7.92, I²: 80%, P: 0.27). This meta-analysis demonstrates that neuromuscular training significantly reduces the incidence of ankle joint injuries, enhances dynamic neuromuscular control, improves foot and ankle function in sports, and appears to positively influence postural stability and ankle instability. However, further high-quality, large-scale RCTs are needed to confirm the preventive effects of neuromuscular training on ankle joint injuries. No external funding was received for this study, and the authors declare no conflicts of interest.

**Systematic review registration:**

https://www.crd.york.ac.uk/PROSPERO/view/CRD42024572932, identifier CRD42024572932.

## 1 Introduction

Ankle joint injuries are a common clinical sports injury, accounting for approximately 18% of all sports-related injuries, making them a significant concern in the field of sports medicine (Allois et al., 2021). These injuries primarily occur due to inadequate warm-up, intense physical activity, or accidental falls, leading to excessive inversion or eversion of the ankle joint (Anguish and Sandrey, 2018). Furthermore, there is a risk of developing long-term sequelae following an ankle injury, such as chronic ankle instability (CAI), talar osteochondral lesions, and persistent residual damage to the joint (Asl et al., 2022). Studies indicate that about 40% of individuals who suffer an ankle injury may eventually develop CAI (Cain et al., 2017). CAI can be categorized into functional instability, characterized by a subjective sense of joint instability and recurrent ankle joint injuries, and mechanical instability, which is caused by ligamentous laxity resulting from ligament injuries (Cain et al., 2020).

Ankle joint injuries can occur across various sports, with football, basketball, and badminton being the most common (Cloak et al., 2013). ankle joint injuries limit an athlete’s ability to run, jump, and change direction, and are typically associated with ligament damage. Due to the close connection and attachment between ligaments, joint capsules, and tendons, injuries often extend to these structures as well (Cumps et al., 2007). Once an ankle injury occurs, there is a heightened risk of reinjury within 12 to 24 months (Cumpston et al., 2019). This recurrent injury is associated with diminished neuromuscular control, delayed peroneal muscle response, reduced strength in the ankle’s inversion and eversion movements, and decreased plantarflexion and dorsiflexion muscle strength (Eils et al., 2010). Such injuries not only incur substantial treatment costs but also increase the risk of early retirement for athletes due to injury (Geist et al., 2021).

To prevent both initial ankle joint injuries (primary prevention) and recurrent sprains (secondary prevention), various interventions and functional training methods have been proposed by physicians, rehabilitation specialists, coaches, and other experts. These interventions include pharmacological treatments (such as the use of non-steroidal anti-inflammatory drugs), bracing (using ankle braces or supports to provide additional stability and protection), heat therapy (applying heat to relax muscles and promote blood circulation), and functional training (such as neuromuscular training) (Gribble et al., 2013).

The athletes were defined as individuals who engage in regular sports activities, while non-athletes were defined as those who do not participate in such activities. Neuromuscular training refers to exercises aimed at improving coordination, strength, and stability through targeted muscle activation (e.g., balance exercises, resistance training). Foot-ankle capability measurements include tests like the Star Excursion Balance Test (SEBT), which assesses dynamic balance and proprioception.

Neuromuscular training is a method that integrates the nervous and muscular systems to enhance coordination, strength, stability, and function. It typically involves a series of targeted exercises designed to improve the connection between the nervous system and muscles, thereby enhancing athletic performance and reducing injury risk. In the context of preventing or treating ankle joint injuries, neuromuscular training often includes exercises such as resistance band training, balance board exercises, and agility ladder drills (Gribble et al., 2016a).

Recent randomized controlled trials (RCTs) have shown that 4–8 weeks of neuromuscular training can effectively improve neuromuscular control in patients with chronic ankle instability, promoting the recovery of proprioception and balance function, thereby reducing the incidence of ankle joint injuries (Gribble et al., 2016b).

However, the research results regarding the efficacy of neuromuscular training on ankle joint injuries have been inconsistent. For instance, P. Y. Huang (Han et al., 2015) found that neuromuscular training did not significantly improve proprioceptive sensitivity in patients with functional ankle instability, nor did it effectively reduce the incidence of ankle joint injuries. Similarly, G. Vuurberg et al (Herzog et al., 2019). reported that neuromuscular and balance training was only effective in preventing recurrent sprains, with no significant impact on preventing the initial occurrence of ankle joint injuries, leaving the therapeutic effects still unclear. Furthermore, ankle joint injuries are influenced by various factors, such as dynamic balance control and foot-ankle capability, which are known predictors of sprains. Attached, Foot-ankle capability refers to the functional capacity of the foot and ankle complex to support and facilitate movement. It includes strength, flexibility, proprioception, and coordination, enabling the ankle to absorb shock, stabilize the body, and maintain proper posture during dynamic activities, which helps prevent injuries like sprains. However, there is currently no comprehensive assessment of these factors in the literature to determine which are most effective in reducing ankle sprain risk. This highlights the need for a new systematic review to scientifically validate these factors through existing research. Therefore, to prevent both initial ankle joint injuries and recurrent sprains, various interventions and functional training methods have been proposed. However, the specific exercises and the optimal dosage of neuromuscular training (e.g., frequency, duration, intensity) are not yet well established, highlighting the need for further investigation into effective protocols.

## 2 Materials and methods

### 2.1 Protocol and registration

This meta-analysis adheres to the guidelines provided by the Preferred Reporting Items for Systematic Reviews and Meta-Analyses (PRISMA) statement (Hertel, 2002). The study has been registered in the PROSPERO database under registration number CRD42024572932.

### 2.2 Search strategy

Two researchers independently conducted the literature search for relevant articles. The detailed search strategy is provided in **Supplementary Materials 1**. The literature search covered publications from the inception of each database until July 31, 2024, and was restricted to English-language articles. The search was conducted across five electronic databases: Cochrane Library, PubMed, Web of Science, Google Scholar, and Embase. To enhance the comprehensiveness and accuracy of the search, tailored search strategies were developed for each database according to its specific functionalities (Hertel and Corbett, 2019). Key search terms included “ankle injury “, “ankle sprain “,”neuromuscular training “,”proprioceptive training “,”balance training “ and “resistance band training “.

### 2.3 Eligibility criteria

Each database was independently searched by two investigators. The researchers independently reviewed the titles, abstracts, and full-text articles retrieved from the search based on the following inclusion and exclusion criteria. In cases of disagreement, a third researcher was consulted to discuss and reach a consensus. Information on risk of bias and other relevant data were collected, and when necessary, contact was made with the study authors to ensure data completeness (Higgins et al., 2019).

### 2.4 Inclusion and exclusion criteria

#### 2.4.1 Inclusion criteria

(1) Study Type: Randomized controlled trials published in English; (2) Participants: Athletes with or without a history of ankle joint injuries, aged 14 years or older, regardless of gender or nationality; (3) Intervention: The experimental group received neuromuscular training, while the control group received no other intervention or only conventional training (Hiller et al., 2006); (4) Primary Outcomes: Incidence of ankle joint injuries, dynamic neuromuscular control, and foot-ankle capability measurements; (5) Secondary Outcomes: Postural stability, joint position sense;

#### 2.4.2 Exclusion criteria

(1) Non-randomized controlled trials, such as reviews, cohort studies, or cross-sectional studies; (2) Studies where the intervention or primary outcomes do not meet the inclusion criteria; (3) Theses or dissertations; (4) Studies involving non-athlete participants; (5) Studies in which neuromuscular training was combined with external co-interventions (e.g., bracing, taping, pharmacological treatment, or manual therapy) and where the independent effect of neuromuscular training could not be determined were excluded; (6) Studies with incomplete data that cannot be retrieved; (7) Duplicate publications;

### 2.5 Study selection

Titles, abstracts, and full-text articles identified through the search were screened according to the inclusion and exclusion criteria by two independent reviewers, Hexuan Xiang and Hyun-Chul Jeong. Any disagreements or differences in opinion were resolved by researchers, Kexiang Yang, Huiling Liao, Yunong Zhang.

### 2.6 Data extraction

Two researchers independently screened the retrieved literature and extracted data using EndNote21. 3 software. The extracted data were then cross-checked to ensure accuracy. In cases of disagreement, discussions were held to resolve the issue, or a third researcher was consulted if necessary to reach a consensus. The extracted data included: first author, publication year, country, study type, study groups, population type, gender, age, sample size, method of diagnosing chronic ankle instability, intervention details, assessment time points, outcome measures, adverse events, and study conclusions (Huang et al., 2014). The quality of the final included studies was assessed using the Cochrane Handbook, and a meta-analysis was conducted using RevMan5.3 software.

### 2.7 Outcome measures

The primary outcome measures were the incidence of ankle joint injuries, SEBT, and foot-ankle capability scores. Secondary outcome measures included ankle postural stability, joint position sense, ankle dorsiflexion range of motion, and Cumberland Ankle Instability Tool scores.

### 2.8 Quality assessment of included studies

The studies included in this analysis were all randomized controlled trials. The quality of the final included studies was assessed using the Cochrane Handbook. Two researchers utilized RevMan5.3 software to evaluate the studies based on seven criteria: random sequence generation, allocation concealment, blinding of participants and personnel, blinding of outcome assessment, completeness of outcome data, selective reporting, and other sources of bias. A quality assessment graph was generated based on these criteria. The risk of bias for each study was determined according to its score: studies scoring 5–7 points were categorized as low risk of bias, those scoring 3–4 points as moderate risk of bias, and those scoring 1–2 points as high risk of bias (Huang et al., 2021). Any differences in assessment between the researchers were resolved through discussion among three reviewers.

### 2.9 Quality of evidence

We used the Grading of Recommendations, Assessment, Development, and Evaluation (GRADE) guidelines to assess the strength of the evidence for each outcome. Since we included RCTs in this meta-analysis, we determined the quality of evidence based on the five domains: risk of bias, inconsistency, indirectness, imprecision, and publication bias. The certainty of evidence was categorized as high, moderate, low, or very low (Hupperets et al., 2009).

### 2.10 Statistical analysis

If the included studies met the criteria for a meta-analysis, data analysis was conducted using RevMan5.3 software. Following the guidelines of the Cochrane Handbook version5.1.0, mean difference (MD) was used as the effect size for continuous variables, and risk ratio (RR) was used as the effect size for dichotomous variables. Each effect size was presented with its corresponding estimate and 95% confidence interval (CI). Heterogeneity was assessed using the chi-square and I2 statistic. A fixed-effects model was applied when P≥0.1 and I²≤ 50%, indicating low heterogeneity (Janssen et al., 2016). If P < 0.1 or I²> 50%, substantial heterogeneity among studies was assumed, and a random-effects model was used. And sensitivity analyses and subgroup analyses were performed to identify the sources of heterogeneity. Sensitivity analysis was conducted by sequentially excluding individual studies to assess their impact on the overall effect size. When more than nine studies were included for a single outcome, a funnel plot was generated to assess publication bias. Statistical significance was set at P < 0.05.

## 3 Results

### 3.1 Literature search results

**Figure 1:**
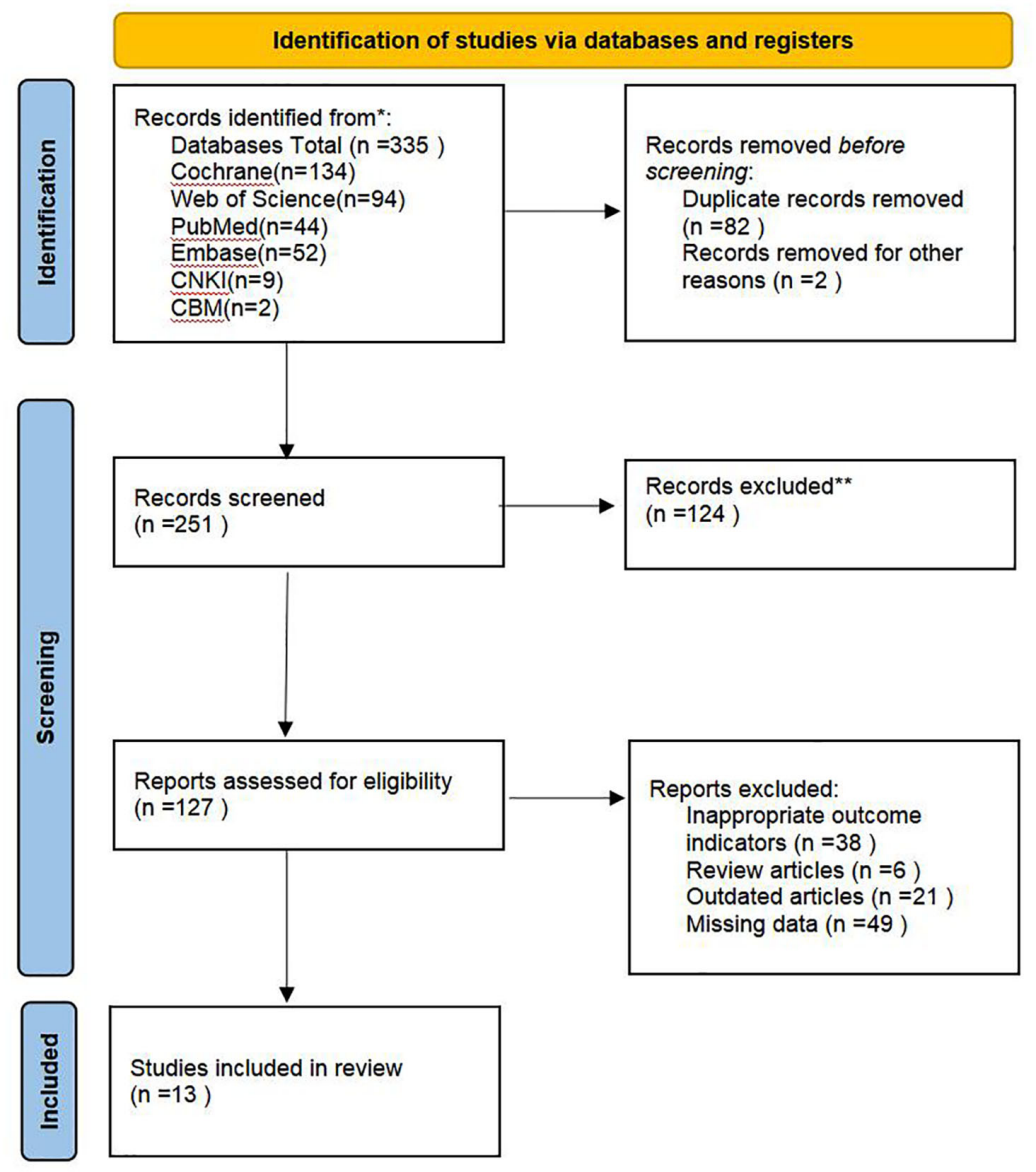
Flow diagram showing selection process of articles following PRISMA guidelines.

A total of 335 articles were initially identified through the literature search, with no additional articles obtained through other sources. After using EndNote21.3 software to remove 82 duplicate articles and excluding 2 review, systematic review, or meta-analysis articles, 251 articles remained. Following an initial screening of titles and abstracts, ultimately, 13 randomized controlled trials involving a total of 1,476 patients were included in the analysis (**Figure 1**).

### 3.2 Characteristics of included studies

This study included 13 articles, encompassing a total of 1,476 participants, with a mixed-gender composition and ages ranging from 14 to 39 years. The interventions were categorized into the following types: (1) balance training;(2) neuromuscular training;(3) resistance band training;(4) traditional single-leg balance training;(5) wobble board balance training;(6) isolated training; and (7) combined training. In this review, “combined training” referred to combinations of exercise modalities within neuromuscular training programs (e.g., balance, proprioceptive, agility, or resistance exercises) and did not include external co-interventions such as bracing, taping, or other therapeutic treatments. There were some variations in the intervention protocols across the studies. First, the duration of interventions ranged from 15 to 45 minutes, with most studies concentrating on a 30-minute session. Second, the frequency of interventions varied from 1 to 7 times per week. Lastly, the intervention periods differed significantly, with the shortest duration being 4 weeks and the longest extending up to 35 weeks. In terms of ankle injury assessment, the primary measurement tools used were the Star Excursion Balance Test, Foot and Ankle Ability Measure, and Cumberland Ankle Instability Tool. The majority of participants were male athletes, with soccer being the most common sport represented. The basic characteristics of the included studies are summarized in **Table 1**.

**Table 1:** Characteristics of studies included for review.

Study	Region	Participant characteristics and Interventions	Sports	frequency	period	Outcomes
Ben Anguish (Anguish and Sandrey, 2018)	U. S.	IG: n: 9, 18. 44 ± 1. 9, Male and female, Traditional single-limb balance training	Comprehensive	3/week; 30minutes	4weeks	FAAM; SEBT
		CG: n: 9, 18. 33 ± 1. 87, Male and female, PHSB training				
Cloak (Cloak et al., 2013)	U. K.	IG: n: 11, 22. 7 ± 1. 2, Male, Balance training	Soccer	2/week	6weeks	SEBT; SLTHD; COM
		CG: n: 11, 23. 1 ± 1. 1Male, No intervention				
Cynthia J (Wright et al., 2017)	U. K.	IG: n: 20, 22. 60 ± 5. 9, Male and female, Wobble-board balance training	Comprehensive	3/week	4weeks	FAAM; SEBT
		CG: n: 20, 21. 45 ± 3. 24, Male and female, Training of resistance-ropes				
Eils (Eils et al., 2010)	Germany	IG: n: 81, 22. 6 ± 6. 3, Male and female, Balance training	Basketball	1/week, 20minute	6weeks	Sense of joint position; Postural sway, Incidence of injuries
		CG: n: 91, 25. 5 ± 7. 2Male and female, No intervention				
Huang	Taiwan	IG: n: 10, 23. 8 ± 4. 1, Male and female, Plyometric training + balance	Comprehensive	3/week	6weeks	Postural sway, SLDL
		CG: n: 10, 23. 5 ± 3. 0, Male and female, No intervention				
Hupperets (Hupperets et al., 2009)	Netherlands	IG: n: 256, 28. 6 ± 11. 8, Male and female, Usual Training +Balance	Comprehensive	3/week, 30minutes	8weeks	Incidence of injuries
		CG: n: 266, 28. 0 ± 11. 6, Male and female, No intervention				
Jessica L	U. S.	IG: n: 13, 18. 4 ± 5. 9, Male and female, Balance training	Comprehensive	2/week 45minutes	4weeks	FAAM; SEBT
		CG: n: 11, 17. 1 ± 0. 3, Male and female, Sham training				
Kidgell (Kidgell et al., 2007)	Australia	IG: n: 10, 25. 4 ± 4. 2, Male and female, Balance training	Comprehensive	3/week	6weeks	Postural sway, COP
		CG: n: 10, 25. 4 ± 4. 2, Male and female, No intervention				
Kyung-Min	Republic of Korea	IG: n: 23, 27. 1 ± 5. 8, Male and female, Neuromuscular training	Comprehensive	2/week	8weeks	FAAM; SEBT; CAIT
		CG: n: 22, 27. 4 ± 8. 3, Male and female, No intervention				
M. Spencer	U. S.	IG: n: 12, 16. 42 ± 1, Male and female, Resistance band training	Comprehensive	3/week	4weeks	FAAM; SEBT; CAIT
		CG: n: 11, 16. 45 ± 1. 04, Male and female, No intervention				
Mohammadi Mohammadi (2007)	Iran	IG: n: 20, 26. 4 ± 2. 6, Male, Balance training	Soccer	7/week, 30minutes	1season	Incidence of injuries
		CG: n: 20, 26. 4 ± 2. 6, Male, No intervention				
Oluwatoyosi	Nigeria	IG: n: 212, 17. 8 ± 0. 9, Male, Neuromuscular training	Soccer	2/3week	6months	Incidence of injuries
		CG: n: 204, 17. 49 ± 1. 10, Male, Usual Training				
Söderman (Söderman et al., 2000)	Sweden	IG: n: 62, 20. 4 ± 4. 6, Female, Usual Training+ Balance	Soccer	3/week 10-15minutes	8months	Flexibility, postural sway, Incidence of injuries
		CG: n: 78, 20. 5 ± 5. 4Female, No intervention				

SEBT, Star excursion balance test; SLTHD, Single-leg triple hop for distance; COM, center of mass; NMT, Neuromuscular training; PHSB, Proprioceptive Hemodynamic Sensory Balance; CAI, Cumberland Ankle Instability; CAIT, Cumberland Ankle Instability Tool; SLDL, Single Leg drop landing; COP, center of pressure; FAAM, Foot and Ankle Ability Measure; IG, intervention group; CG, control group.

### 3.3 Quality assessment results of included studies

**Figure 2:**
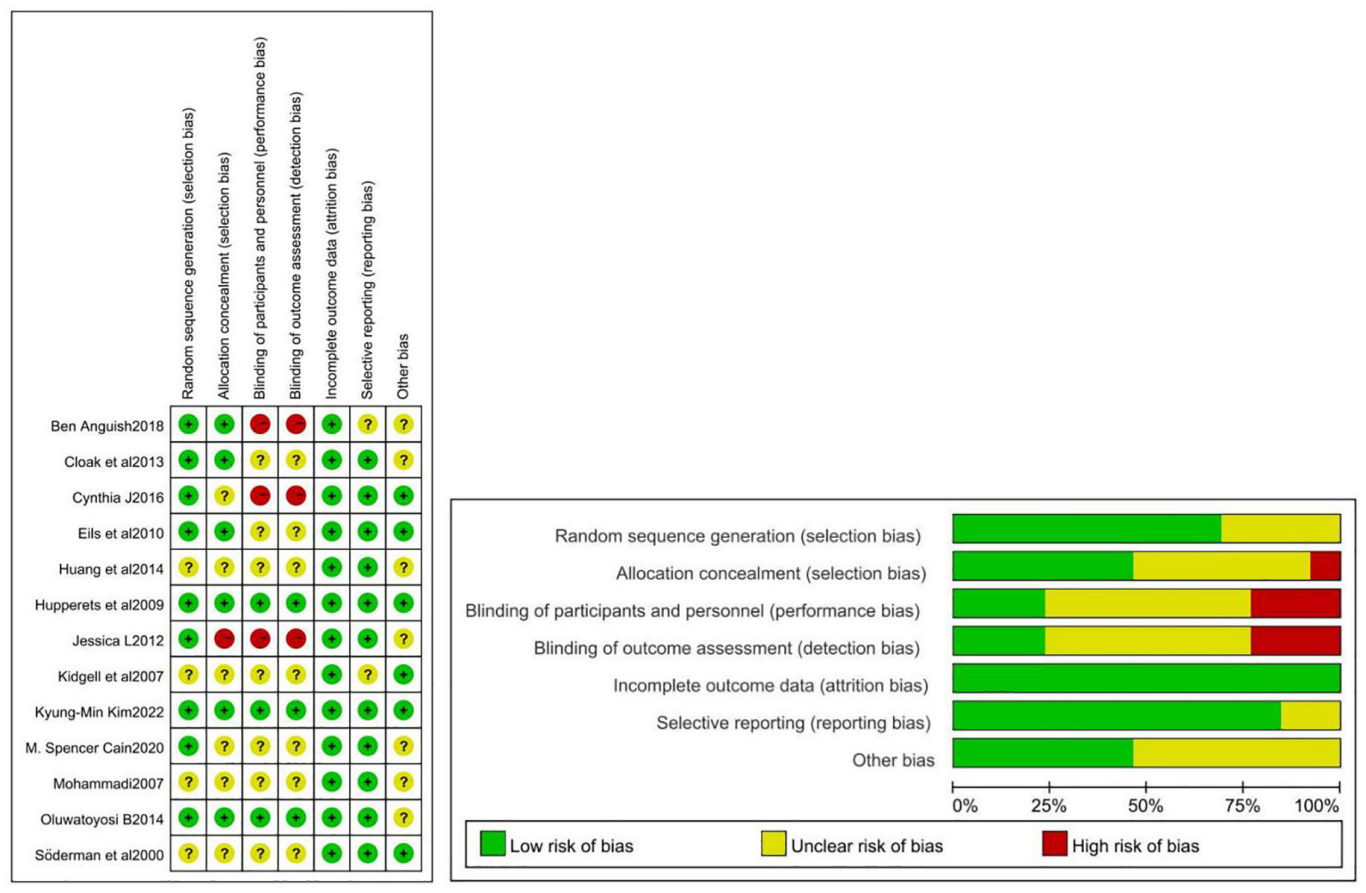
Risk of bias graph and summary.

The methodological quality of the 13 included studies was evaluated using the Cochrane risk of bias assessment tool, focusing on seven key areas: random sequence generation, allocation concealment, blinding of participants and personnel, blinding of outcome assessment, incomplete outcome data, selective reporting, and other biases. As illustrated in **Figure 2**, out of the 13 studies, 6 were rated as Grade A, 7 as Grade B, and none as Grade C.

### 3.4 Incidence of ankle joint injuries

**Figure 3:**
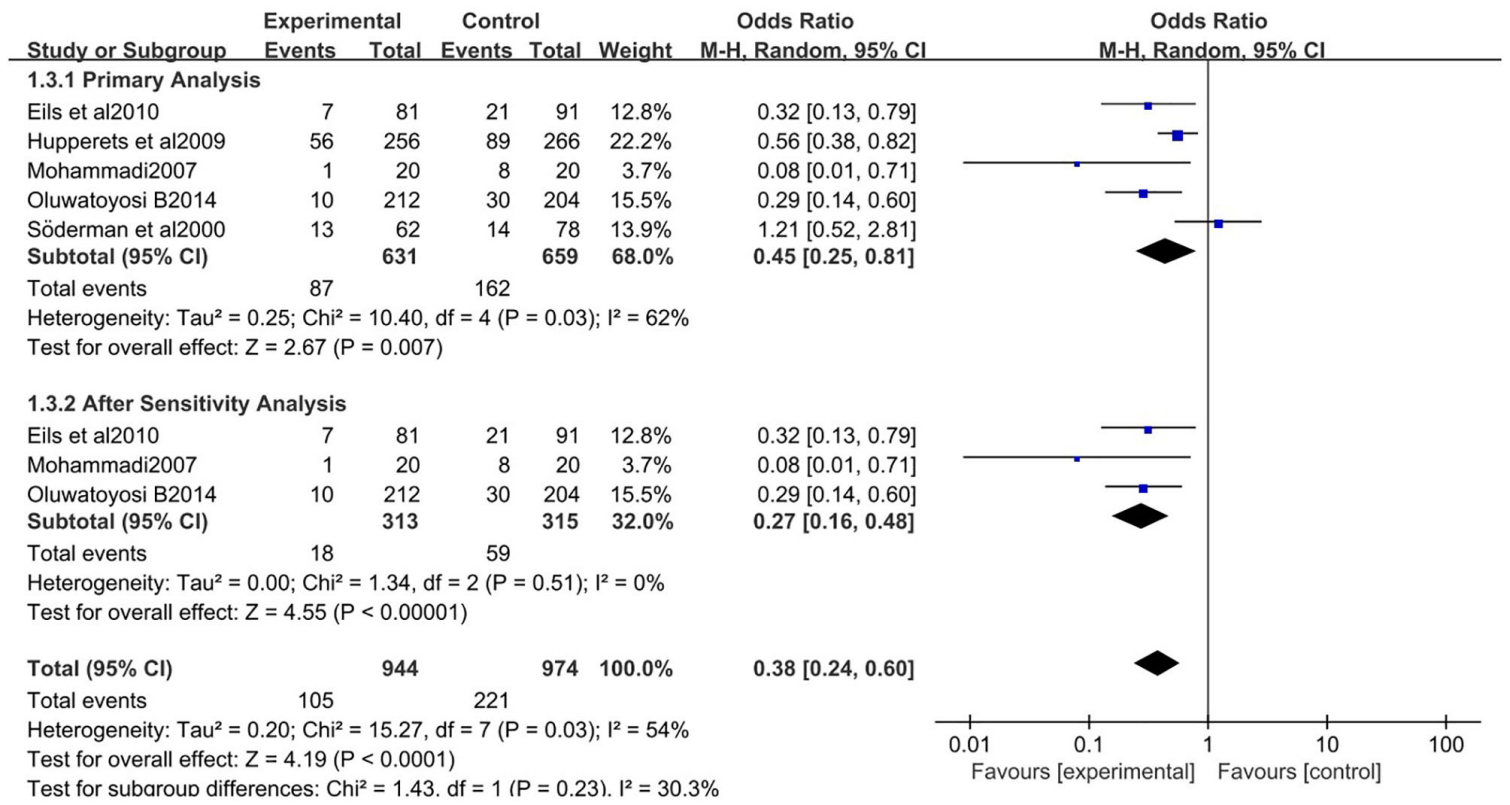
Meta-analysis forest plots showing relative risk of ankle sprain.

Five studies (n=1,290) included in this systematic review examined the incidence of ankle joint injuries (Higgins et al., 2003; Kidgell et al., 2007; Kalirathinam et al., 2018a; Kalirathinam et al., 2018b; Khalili et al., 2022), making them eligible for meta-analysis. Compared to the control group, the neuromuscular training group showed a 55% reduction in the incidence of ankle joint injuries (RR: 0.45, 95%CI: 0.25-0.81, I²: 62%, P: 0.007). Due to observed inconsistency in the results, a sensitivity analysis was conducted, revealing that the inconsistency was related to the type of intervention. The studies by Hupperets and Söderman were excluded in the sensitivity analysis because their intervention protocols consisted of regular sport-specific training (conventional training) supplemented with balance training exercises. Conventional training included routine technical, tactical, and conditioning activities, whereas balance training comprised neuromuscular exercises targeting proprioception and postural control. This intervention structure differed from that of most included studies and was considered a potential source of heterogeneity. After their exclusion, the heterogeneity dropped to 0, with no change in the results (RR: 0.27, 95%CI: 0.16-0.48, I²: 0%, P: <0.00001) (**Figure 3**).

### 3.5 Dynamic neuromuscular control

**Figure 4:**
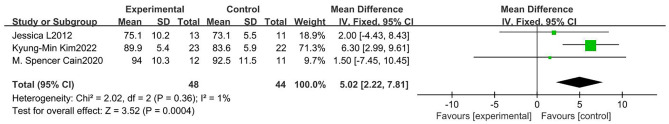
Forest plots showing the relative distance in the posteromedial direction in SEBT.

**Figure 5:**
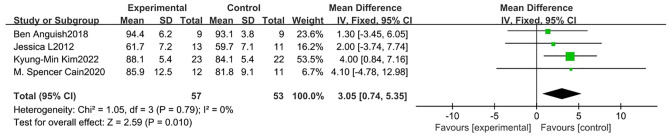
Forest plots showing the distance in the posterolateral direction in SEBT.

**Figure 6:**
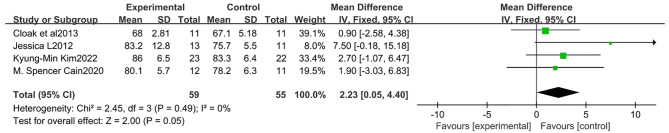
Forest plots showing the distance in the anterior direction in SEBT.

In the systematic review, five studies (n=132) (McKay et al., 2001; Linford et al., 2006; Kim et al., 2022; Lee et al., 2022; Liu, 2022) used the Star Excursion Balance Test to assess dynamic neuromuscular control. Data from three studies (Kim et al., 2022; Lee et al., 2022; Liu, 2022) were suitable for meta-analysis, as they recorded the same measurement directions: posteromedial, posterolateral, and anterior (**Figures 4**-**6**). The analysis showed that balance training led to significant increases in reach distance in the posteromedial (MD: 5.02 cm, 95%CI: 2.22-7.81, I²: 1%, P: 0.0004) (**Figure 4**), posterolateral (MD: 3.05 cm, 95%CI: 0.74-5.35, I²: 0%, P: 0.01) (**Figure 5**), and anterior (MD: 2.23 cm, 95%CI: 0.05-4. 40, I²: 0%, P: 0.05) (**Figure 6**) directions.

### 3.6 Foot and ankle ability measure

#### 3.6.1 Activity daily living

**Figure 7:**
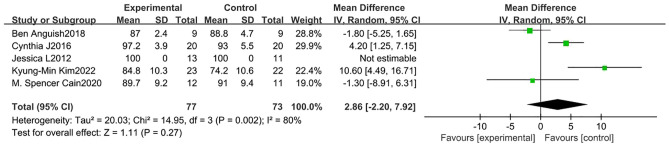
Forest plots showing the Foot and Ankle Ability Measure - ADL.

Five studies involving 150 participants (McKay et al., 2001; Linford et al., 2006; Moher et al., 2009; Kim et al., 2022; Lee et al., 2022) reported on the impact of neuromuscular training on foot and ankle ability in activity daily living. The meta-analysis showed no significant difference between the experimental group and the control group (MD: 2.86, 95%CI: -2.20 to 7.92, I²: 80%, P: 0.27), as the confidence interval crossed zero (**Figure 7**).

#### 3.6.2 Sport

**Figure 8:**
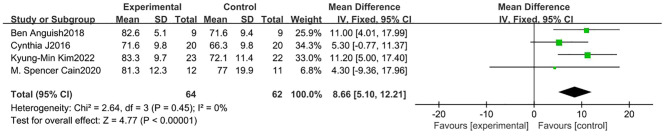
Forest plots showing the Foot and Ankle Ability Measure - sport.

Four studies involving 126 participants (Linford et al., 2006; Moher et al., 2009; Kim et al., 2022; Lee et al., 2022) reported on the impact of neuromuscular training on foot and ankle ability in sports activities. The meta-analysis revealed a significant difference between the experimental group and the control group (MD: 8.66, 95%CI: 5.10-12.21, I²: 0%, P: <0.00001) (**Figure 8**).

### 3.7 Postural sway

Two studies (Mohammadi, 2007; Mulla and Keir, 2023) reported on the effects of proprioceptive training on postural stability in athletes. The evaluation tools used included force platforms and motion capture system balance testers. Due to significant differences in the measurement tools, a meta-analysis was not feasible, so only a descriptive analysis was conducted. The results of these two studies indicated that athletes in the experimental group showed significant improvement in the medial direction after balance training, such as reduced sway. However, no significant differences were observed in other directions.

### 3.8 Results measured by the Cumberland Ankle Instability Tool

Only two studies reported CAIT scores (Kim et al., 2022; Lee et al., 2022), which was insufficient for a meta-analysis, so a descriptive analysis was conducted instead. The findings from these studies showed that athletes in the experimental group, after undergoing neuromuscular and resistance band training, had significantly improved CAIT scores, while the control group showed no significant changes.

### 3.9 Certainty of evidence

The GRADE system evaluated the certainty of evidence for the impact of neuromuscular training on preventing ankle joint injuries, with scores ranging from low to moderate (13 RCTs included). For the relative risk of ankle joint injuries, the evidence was rated as moderate quality (RR: 0.27, 95%CI: 0.16-0.48). The evidence for the SEBT in the posteromedial direction was rated as low quality (MD: 5.02, 95%CI: 2.22-7.81). The evidence for the SEBT in the posterolateral direction was also rated as low quality (MD: 3.05, 95%CI: 0.74-5.35). Similarly, the evidence for the SEBT in the anterior direction was rated as low quality (MD: 2.23, 95%CI: 0.05-4.40). For the FAAM-Daily Living, the evidence was rated as moderate quality (MD: 2.86, 95%CI: -2.20-7.92). For the FAAM-Sport, the evidence was also rated as moderate quality (MD: 8.66, 95%CI: 5.10-12.21).

## 4 Discussion

This systematic review and meta-analysis provided a comprehensive analysis and interpretation of the effects of neuromuscular training, confirming its efficacy in reducing the incidence of ankle joint injuries, enhancing dynamic neuromuscular control across various sports, and improving athletes’ perceived foot and ankle performance in sports activities. Additionally, the systematic review indicated that neuromuscular training slightly improved postural sway and ankle stability in athletes. However, it remains inconclusive regarding its impact on perceived foot and ankle performance in daily living activities. The incidence of injury is a crucial clinical indicator of ankle health, and the risk of sports injuries is closely related to factors such as neuromuscular control, postural sway, strength, and balance (Munn et al., 2010).

Regarding the reduction in ankle sprain incidence among athletes, ankle joint injuries are a common sports injury with high initial and recurrent injury rates, with recurrence rates for ankle joint injuries in athletes reaching as high as 70% to 80% (Nasser, 2020). Following a sprain, an athlete’s quality of life, as well as their daily training and competition, can be affected to varying degrees. The results of this meta-analysis showed that the incidence of ankle joint injuries in the experimental group was significantly lower than in the control group. This suggests that neuromuscular training can enhance ankle stability, thereby reducing the incidence and recurrence of ankle joint injuries in athletes. In the study by Konstantinos Tsikopoulos et al (Neto et al., 2022), they summarized 26 high-quality trials and concluded that balance board training could reduce the risk of recurrent ankle joint injuries by 54%, consistent with the studies (Owoeye et al., 2014). Several potential mechanisms have been proposed in previous studies, including enhanced proprioceptive input and improved sensorimotor integration. However, the current review did not directly evaluate these mechanisms, and therefore such explanations remain speculative (Propadalo et al., 2019). Moreover, neuromuscular training may improve the coordination of the muscles surrounding the ankle during various athletic activities through feedback from muscles and tendons. Additionally, the increased muscle contraction rate and reaction time also contribute to better control of the ankle joint (Robinson and Dickersin, 2002).

### 4.1 Regarding the improvement of athletes’ neuromuscular control

Neuromuscular control refers to the precise muscle activation process that enables the human body to produce coordinated and effective movements during physical activity, primarily relying on the proper functioning of the sensorimotor system (Schaefer and Sandrey, 2012). The Star Excursion Balance Test is an important tool for assessing neuromuscular function, with test results demonstrating high reliability (Sefton et al., 2011). The meta-analysis results of this study showed that the total SEBT scores for the experimental group were significantly higher than those of the control group, with notable improvements in the maximum reach distances in the posterolateral, posteromedial, and anterior directions. These findings suggest that neuromuscular training may improve reach distance in the posteromedial, posterolateral, and anterior directions; however, the certainty of evidence was rated as low and the results should therefore be interpreted with caution. These findings are consistent with the results of Abed Taghavi Asl et al (Sierevelt et al., 2024). Several potential mechanisms have been proposed in previous studies, including enhanced proprioceptive input and improved sensorimotor integration. However, the current review did not directly evaluate these mechanisms, and therefore such explanations remain speculative. Furthermore, it may also improve coordination in resisting external disturbances and agility in movement.

### 4.2 Regarding foot and ankle ability in daily living/sports

Foot and ankle ability is assessed using a questionnaire tool designed to evaluate the function of the ankle and foot. The Foot and Ankle Ability Measure scale is primarily used to assess an individual’s functional status following foot and ankle joint injuries or disorders (Söderman et al., 2000). In the Activities of Daily Living section, which focuses on the functional performance of the ankle in daily activities such as walking and climbing stairs, the meta-analysis results indicated no statistically significant differences between the control and experimental groups. Further investigation into improving ADL is needed. The Sports and Work section emphasizes evaluating an individual’s functional performance in sports or work settings, particularly in activities requiring higher intensity or specialized motor skills (Tsikopoulos et al., 2018). In the analysis of Sports and Work, the total scores of the experimental group were significantly higher than those of the control group, indicating marked improvements in this area, consistent with the findings of D. Kalirathinam et al (Vuurberg et al., 2018). The mechanism behind this improvement may lie in the fact that neuromuscular training strengthens the muscle groups surrounding the ankle joint, enhancing their ability to support the joint. However, despite the observed improvements in dynamic balance and sport-related ankle function, no significant improvement was observed in FAAM-ADL scores. One possible explanation is that most participants were athletes with relatively high baseline levels of daily functional performance, resulting in limited room for further improvement. In addition, the FAAM-ADL primarily assesses basic daily activities, whereas neuromuscular training may exert greater effects on sport-specific tasks requiring rapid postural adjustments, proprioceptive control, and dynamic stability. Consequently, improvements in balance performance may not necessarily translate into measurable gains in daily living activities. Stronger muscles not only better protect the joint from injury but also contribute to improved overall athletic performance.

### 4.3 Regarding postural sway

Balance training optimizes certain aspects of postural control, enhancing the efficiency of incoming and outgoing neural signal transmission. However, according to H. Lee’s study (Wright and Linens, 2017), no differences were observed in any direction post-training. This result could be due to the relatively short duration of the training program, which lasted only 4 weeks, compared to the 6-week span used in Huang et al. ‘s study, making it difficult to detect significant changes in a shorter timeframe. Therefore, Limited evidence from two studies suggested that neuromuscular training may improve postural sway and ankle stability in athletes; However, these findings should be interpreted cautiously because the evidence was based on a small number of studies and was not suitable for quantitative synthesis.

### 4.4 Regarding CAIT scores

This systematic review shows that the experimental group had significantly higher CAIT scores than the control group. The Cumberland Ankle Instability Tool is a multidimensional, comprehensive scale for assessing chronic ankle instability (Wright et al., 2017), encompassing aspects such as ankle pain, subjective feelings of instability during daily and physical activities, and the ankle’s response to pain (51). As an auxiliary scale, the CAIT may provide valuable insights into ankle health status. The results suggest that neuromuscular training improves CAIT scores; however, due to the limited number of related studies, determining the preventive effects of neuromuscular training on ankle health solely based on CAIT scores remains uncertain.

## 5 Limitations and future research directions

This study has several limitations. First, the variability in the types of neuromuscular training, participant age, and competitive levels among the included studies increased heterogeneity. Second, the nature of preventive training makes it difficult to blind participants; most studies also did not implement assessor blinding, and several studies did not clearly report allocation concealment, which may introduce bias. Third, the small sample sizes across various dimensions in the included studies increase the likelihood of Type II errors. Fourth, due to the limited number of studies available for each dimension, it was not possible to conduct publication bias tests, presenting a potential risk of bias. Fifth, The substantial heterogeneity observed for FAAM-ADL outcomes may be related to differences in participant characteristics (e.g., chronic ankle instability history), intervention duration, exercise content, and baseline functional status. Given that only five studies were available, subgroup analyses were not considered statistically robust, and the findings should therefore be interpreted with caution. Finally, the lack of long-term follow-up investigations makes it challenging to assess the long-term efficacy of neuromuscular training in preventing ankle joint injuries. Another limitation is that this review relied on aggregate study-level data rather than individual participant data (IPD). Consequently, we were unable to explore potentially important moderators, such as sex, sport type, age, baseline ankle instability status, or injury history. Future IPD meta-analyses may help identify subgroups that derive the greatest benefit from neuromuscular training and provide more individualized recommendations for injury prevention.

Given the diversity of sports, each requiring different muscle conditions, positions, power application methods, and coordination strategies, the design of neuromuscular training programs should take into account the specific characteristics of the sport. This will enhance the scientific basis, accuracy, and timeliness of interventions aimed at the ankle joint. A systematic and comprehensive neuromuscular training program remains a focus for future research.

## 6 Conclusions

In summary, neuromuscular training was associated with a substantial reduction in the risk of ankle joint injuries and improvements in dynamic neuromuscular control and sport-related foot and ankle function. However, further research is needed to validate its effects on the strength of the muscles surrounding the ankle, proprioception, range of motion, and functional outcome measures. Given the limited number of studies included regarding ankle muscle control in the posteromedial direction, foot and ankle function in daily living and sports activities, postural sway, and CAIT scores, the results should be interpreted with caution. Future researchers should conduct large-scale, high-quality studies to establish the clinical efficacy of neuromuscular training.

## Data Availability

The original contributions presented in the study are included in the article/**Supplementary Material**. Further inquiries can be directed to the corresponding author.
