# Supplementary material for: Neuromuscular training for preventing ankle joint injuries in athletes: a systematic review and meta-analysis
Source: Front Physiol. 2026 Jul 22;17:1854319. doi: 10.3389/fphys.2026.1854319 (PMC13437404; doi:10.3389/fphys.2026.1854319)

Web of science

neuromuscular exercise OR neuromuscular sports OR neuromuscular training OR Functional training OR Proprioceptive training OR Movement efficiency training OR Sensorimotor training OR Balance training OR Core stability training OR proprioceptive OR proprioception OR sensorimotor OR Proprioceptions OR Sense of Position OR proprioceptive balance board training (Topic) and Ankle Injuries OR Ankle Injury OR Syndesmotic Injuries OR Syndesmotic Injury OR Ankle Sprain OR Ankle Sprains OR Tarsus OR Regio tarsalis OR Injury, Ankle OR Injury, Ankle OR Sprain, Ankle OR Sprains, Ankle OR Injuries, Syndesmotic OR Injury, Syndesmotic (Title) and Exercise OR Exercises OR Sports OR Physical Activity OR Motor Activity OR Training OR endurance training OR Resistance OR Aerobic OR Aerobic OR Tai Chi OR yoga OR Balance OR Flexibility OR badminton OR football OR Table Tennis OR high intensity interval training OR athlet OR volleyball OR soccer OR handball OR tennis OR netball OR hockey OR softball OR gymnastics (Topic) and “Randomized controlled trial” OR “controlled clinical trial” OR “RCT” OR “randomized” OR “placebo” OR “randomly” (Abstract)

Pubmed

(((neuromuscular exercise[Title/Abstract] OR neuromuscular sports[Title/Abstract] OR neuromuscular training[Title/Abstract] OR Functional training[Title/Abstract] OR Proprioceptive training[Title/Abstract] OR Movement efficiency training[Title/Abstract] OR Sensorimotor training[Title/Abstract] OR Balance training[Title/Abstract] OR Core stability training[Title/Abstract] OR proprioceptive[Title/Abstract] OR proprioception[Title/Abstract] OR sensorimotor[Title/Abstract] OR Proprioceptions[Title/Abstract] OR Sense of Position[Title/Abstract] OR proprioceptive balance board training[Title/Abstract]) AND (Ankle Injuries[Title/Abstract] OR Ankle Injury[Title/Abstract] OR Syndesmotic Injuries[Title/Abstract] OR Syndesmotic Injury[Title/Abstract] OR Ankle Sprain[Title/Abstract] OR Ankle Sprains[Title/Abstract] OR Tarsus[Title/Abstract] OR Regio tarsalis[Title/Abstract] OR Injury, Ankle[Title/Abstract] OR Injury, Ankle[Title/Abstract] OR Sprain, Ankle[Title/Abstract] OR Sprains, Ankle[Title/Abstract] OR Injuries, Syndesmotic[Title/Abstract] OR Injury, Syndesmotic[Title/Abstract])) AND (Exercise[Title/Abstract] OR Exercises[Title/Abstract] OR Sports[Title/Abstract] OR Physical Activity[Title/Abstract] OR Motor Activity[Title/Abstract] OR Training[Title/Abstract] OR endurance training[Title/Abstract] OR Resistance[Title/Abstract] OR Aerobic[Title/Abstract] OR Aerobic[Title/Abstract] OR Tai Chi[Title/Abstract] OR yoga[Title/Abstract] OR Balance[Title/Abstract] OR Flexibility[Title/Abstract] OR badminton[Title/Abstract] OR football[Title/Abstract] OR Table Tennis[Title/Abstract] OR high intensity interval training[Title/Abstract] OR athlet[Title/Abstract] OR volleyball[Title/Abstract] OR soccer[Title/Abstract] OR handball[Title/Abstract] OR tennis[Title/Abstract] OR netball[Title/Abstract] OR hockey[Title/Abstract] OR softball[Title/Abstract] OR gymnastics[Title/Abstract])) AND ("Randomized controlled trial"[Title/Abstract] OR "controlled clinical trial"[Title/Abstract] OR "RCT"[Title/Abstract] OR "randomized"[Title/Abstract] OR "placebo"[Title/Abstract] OR "randomly"[Title/Abstract])

Embase

(Exercise OR Exercises OR Sports OR Physical Activity OR Motor Activity OR Training OR endurance training OR Resistance OR Aerobic OR Aerobic OR Tai Chi OR yoga OR Balance OR Flexibility OR badminton OR football OR Table Tennis OR high intensity interval training OR athlet OR volleyball OR soccer OR handball OR tennis OR netball OR hockey OR softball OR gymnastics)/br AND (('Ankle Injuries' OR 'Ankle Injury' OR 'Syndesmotic Injuries' OR 'Syndesmotic Injury' OR 'Ankle Sprain' OR 'Ankle Sprains' OR Tarsus OR 'Regio tarsalis' OR 'Injury, Ankle' OR 'Injury, Ankle' OR 'Sprain, Ankle' OR 'Sprains, Ankle' OR 'Injuries, Syndesmotic' OR 'Injury, Syndesmotic'):ti,ab,kw) AND (('neuromuscular exercise' OR 'neuromuscular sports' OR 'neuromuscular training' OR 'Functional training' OR 'Proprioceptive training' OR 'Movement efficiency training' OR 'Sensorimotor training' OR 'Balance training' OR 'Core stability training' OR proprioceptive OR proprioception OR sensorimotor OR Proprioceptions OR 'Sense of Position' OR 'proprioceptive balance board training'):ti,ab,kw) AND ((“ Randomized controlled trial ” OR “ controlled clinical trial ” OR “ RCT ” OR “ randomized ” OR “ placebo ” OR “randomly”):ti,ab,kw)

Cochrane library

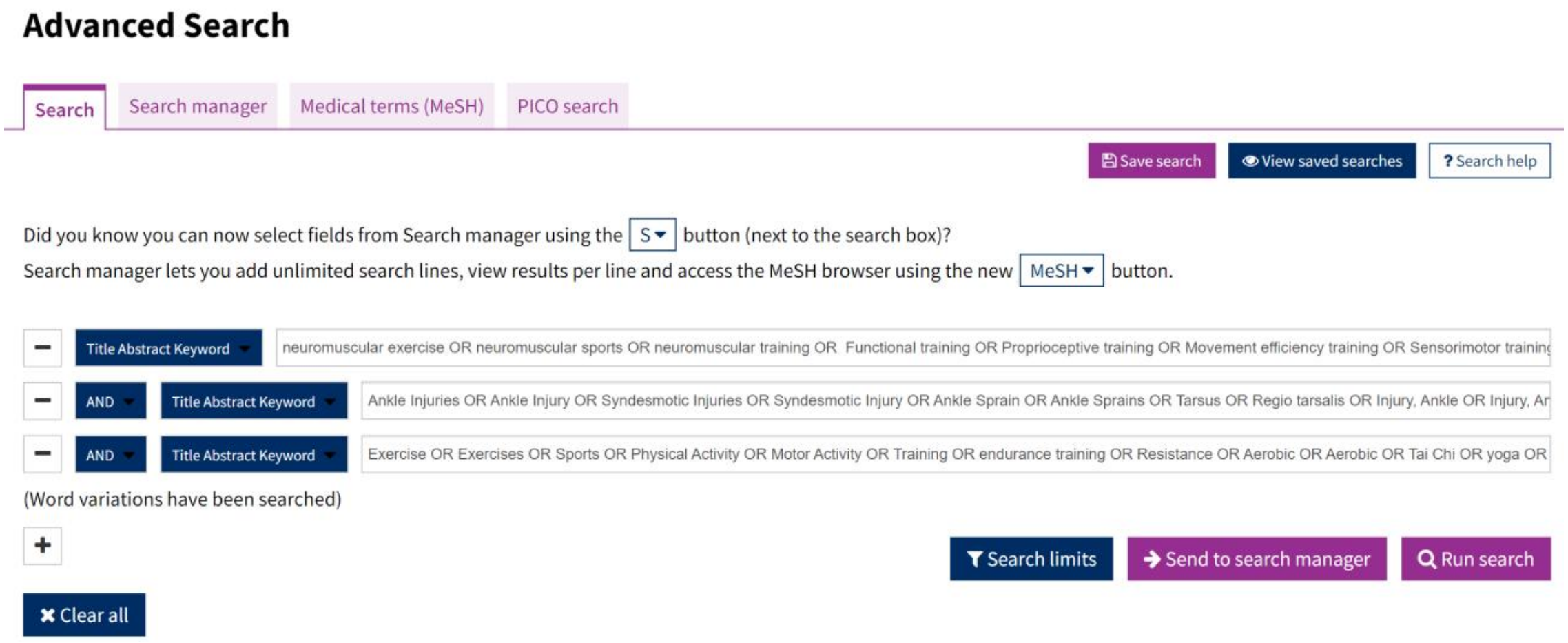

Google Scholar

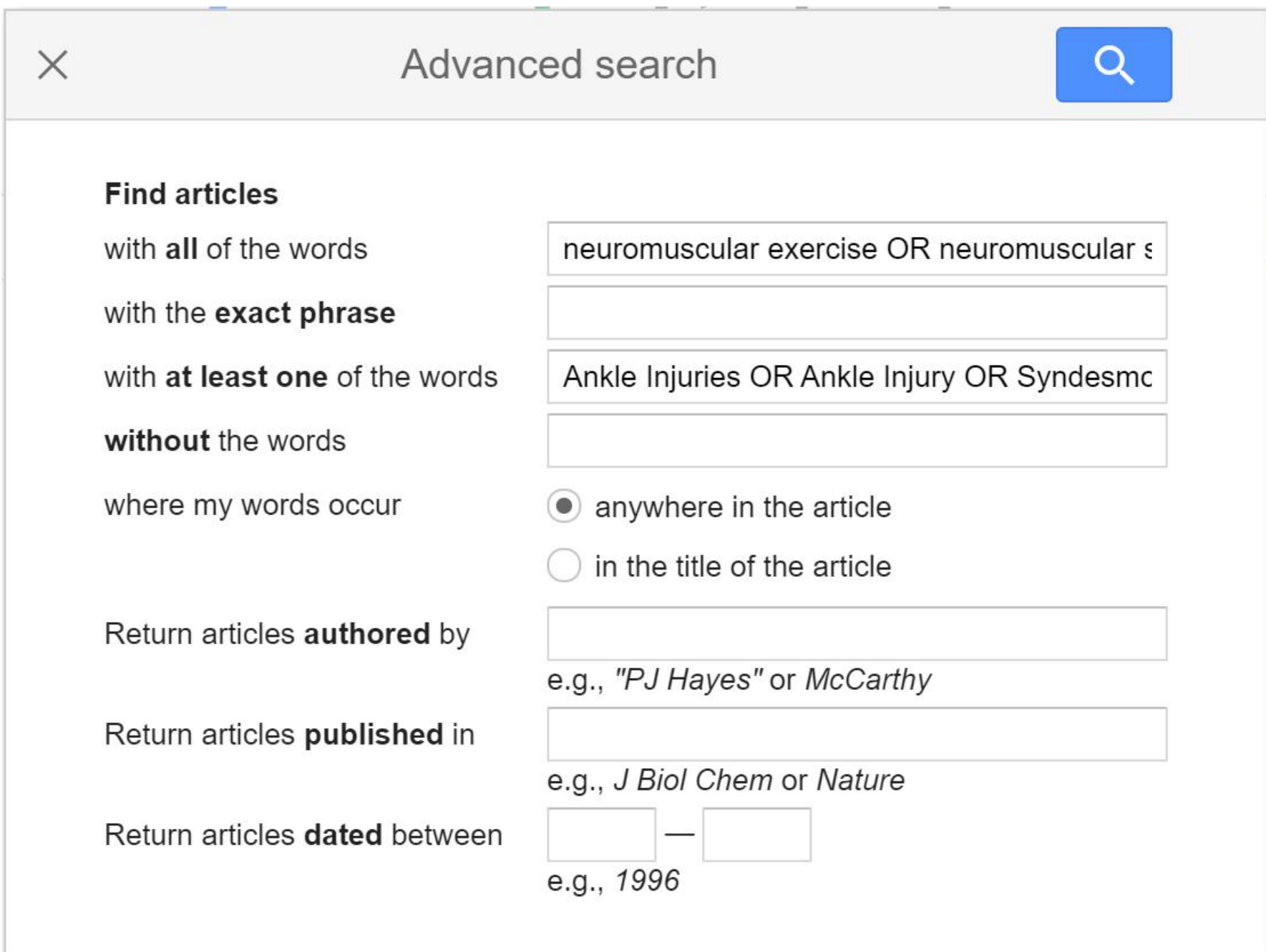

Supplement: Supplementary file 1 [file DataSheet1.pdf]
