# Supplementary material for: Neuromuscular training for preventing ankle joint injuries in athletes: a systematic review and meta-analysis
Source: Front Physiol. 2026 Jul 22;17:1854319. doi: 10.3389/fphys.2026.1854319 (PMC13437404; doi:10.3389/fphys.2026.1854319)
Supplement: Supplementary file 2 [file DataSheet2.pdf]

S3 Table. GRADE Assessment.

Neuromuscular training versus Control

Outcomes	Study Design	Risk of Bias	Inconsistency	Indirectness	Imprecision	Other Considerations	No. of Participants		Absolute Effect (95% CI)	Quality
							NMT	Control		
relative risk of ankle sprain	RCTs	Serious	No	No	No	No	313	315	MD 0.27 (0.16 to 0.48)	Moderate
posteromedial direction in SEBT	RCTs	Serious	No	No	Serious	No	48	44	MD 5.02 (2.22 to 7.81)	Low
posterolateral direction in SEBT	RCTs	Serious	No	No	Serious	No	57	53	MD 3.05 (0.74 to 5.35)	Low
anterior direction in SEBT	RCTs	Serious	No	No	Serious	No	59	55	MD 2.23 (0.05 to 4.40)	Low
FAAM-Daily Living	RCTs	Serious	No	No	No	No	77	73	MD 2.86 (-2.20 to 7.92)	Moderate
FAAM-Sport	RCTs	Serious	No	No	No	No	64	62	MD 8.66 (5.10 to 12.21)	Moderate

RCTs, randomized controlled trials. MD, mean difference. SMD, standardized mean difference. NMT,Neuromuscular training. SEBT,Star excursion balance test
